# Analytical Operations Relate Structural and Functional Connectivity in the Brain

**DOI:** 10.1371/journal.pone.0157292

**Published:** 2016-08-18

**Authors:** Maria Luisa Saggio, Petra Ritter, Viktor K. Jirsa

**Affiliations:** 1 Institut de Neurosciences des Systèmes, Aix-Marseille Université Faculté de Médecine, Marseille, France; 2 INSERM UMR 1106, Aix-Marseille Université, Marseille, France; 3 Minerva Research Group BrainModes, Max Planck Institute for Human Cognitive and Brain Sciences, Leipzig, Germany; 4 Dept. Neurology, Charité - University Medicine, Berlin, Germany; 5 Bernstein Focus State Dependencies of Learning & Bernstein Center for Computational Neuroscience, Berlin, Germany; 6 Berlin School of Mind and Brain & Mind and Brain Institute, Humboldt University, Berlin, Germany; University of Texas at Austin, UNITED STATES

## Abstract

Resting-state large-scale brain models vary in the amount of biological elements they incorporate and in the way they are being tested. One might expect that the more realistic the model is, the closer it should reproduce real functional data. It has been shown, instead, that when linear correlation across long BOLD fMRI time-series is used as a measure for functional connectivity (FC) to compare simulated and real data, a simple model performs just as well, or even better, than more sophisticated ones. The model in question is a simple linear model, which considers the physiological noise that is pervasively present in our brain while it diffuses across the white-matter connections, that is structural connectivity (SC). We deeply investigate this linear model, providing an analytical solution to straightforwardly compute FC from SC without the need of computationally costly simulations of time-series. We provide a few examples how this analytical solution could be used to perform a fast and detailed parameter exploration or to investigate resting-state non-stationarities. Most importantly, by inverting the analytical solution, we propose a method to retrieve information on the anatomical structure directly from functional data. This simple method can be used to complement or guide DTI/DSI and tractography results, especially for a better assessment of inter-hemispheric connections, or to provide an estimate of SC when only functional data are available.

## Introduction

Large-scale brain dynamics evolves spatiotemporally as a network within constraints imposed by its structural connectivity (SC). The link between structure and function has been addressed in many studies and, even though the existence of anatomical wiring between two regions has shown to be a good predictor of correlation activity between those two regions, the inverse is not always true [1–3]. There are many factors that can influence this relationship. An important role is played by characteristics of the structure itself: the length of the white matter fiber tracts and the subsequent time delays in signal transmission among brain areas, which affect their degree of synchronization [4–10]; the modularity of the network [11]; the degree product of its nodes [12] or other characteristics of network communication [13], among other factors. But function does not simply mirror structure and the understanding of functional connectivity (FC), i.e. statistical dependencies in the network activity, calls for an inclusion of the dynamics into the network description. This has been stated succinctly by Deco, Jirsa and McIntosh [14] by writing “The missing link for understanding the formation and dissolution of the resting state networks is the dynamics”. Models for large-scale brain behavior can vary in the amount of biological elements they incorporate and the type of dynamics they display (e.g. linear, non-linear, deterministic, stochastic, chaotic) both at the local and at the global level and can be strongly affected by the values assigned to parameters [15].

Augmenting the realism of a model for brain activity by trying to incorporate complex dynamics and physiological details can improve our understanding of the link between structure and function, but, at the same time, it makes the simulations of the model computationally expensive and reduces the possibility of gaining analytical insights. Thus it is useful to reduce the model depending on the specific question addressed and the nature of the available data as well as measures. For example, when considering firing rates of a network, the temporal aspect of couplings is often neglected [16]. This negligence may be justified under certain conditions, at least for the equilibrium dynamics of the network, but has to be carefully reconsidered when deviations away from the equilibrium occur [17–19].

When modelling the Resting State Network (RSN) dynamics, FC is often used to compare model predictions with empirical data. FC is, generally speaking, the complete set of functional relationships between brain areas and has been quantified using different kinds of measures of statistical dependencies, such as correlations, coherence, mutual information or transfer entropy [20, 21]. A common choice is that of the linear Pearson Correlation Coefficient (PCC) computed for each pair of brain areas over long time-series (of the order of 10 minutes for the BOLD signal) [16, 22], and we will refer to it when mentioning FC unless otherwise specified. PCC, though, relies on the assumption of stationarity of the resting-state data, but, despite the fact that large-scale structural connectivity changes at very slow time scales (in the order of days to years), resting-state FC computed over smaller time windows varies greatly during a scan session [1, 23], possibly due to local plasticity mechanisms [24] or to a changing cognitive state among other factors. Using PCC as a measure for FC does not do justice to the complexity present in the data and in complex non-linear models, in particular is incapable of capturing any non-stationarity of the resting state network (RSN) dynamics [14, 21, 22, 25].

By construction FC quantifies the degree of linear dependence between any two variables and thus only captures second-order moments. For this reason it is not well suited to discriminate the predictive power of non-linear models which have been proposed to describe the dynamics of large-scale brain activity. In fact, when FC is used to assess the performance of models for large-scale brain activity, linear models have shown to perform equally good or even better than more sophisticated and more biologically realistic non-linear ones as recently demonstrated by Messé and collaborators [22]. This result does not imply that the dynamical processes underlying the BOLD signal are indeed linear, for example it is known that both neuronal activity and neuro-vascular coupling are not linear, but points at the necessity of using different paradigms when investigating the performance of models for such activities. Different results are indeed obtained when metrics able to track non-stationarities are considered, such as functional connectivity dynamics (FCD) [26] or other metrics introduced to test the presence of non-stationarities in resting state BOLD human data and their statistical significance [27]. Messé et al. [22] estimated that anatomical connectivity alone accounts for up to 15% of FC variance and stationary FC to about 20%, leaving a large remaining variance (around 65%) to non-stationarities of FC.

Nevertheless, there is a big benefit in applying FC, measured over long time windows, to evaluate network behavior. In particular, it has been suggested that FC may serve as an endophenotype to infer and classify the group to which a given subject belongs [20]. There are now numerous clinical applications of FC, computed also from other imaging modalities such as MEG, including in Alzheimer’s disease, schizophrenia, autism, pre-surgical planning, epilepsy, aging and traumatic brain injury [14, 28–30]. Group analyses show highly statistically significant results, though strong underlying inter-subject and intra-subject variability [31] may preclude accurate individual predictability.

Motivated by the fact the simple models are as good as more sophisticated ones in predicting stationary FC matrices for the BOLD signal and that the analysis of these long time averaged data offer important insights [16, 32] as well as being simple to perform, we aim to use the simplest possible model able to generate stationary dynamics, that is linear diffusion of noise over the anatomical structure, in order to develop analytical methods that are easy and fast applicable.

For their usefulness and for the possibility of using analytical approaches, linear models have been already introduced in the context of the structure-function relationship. One of the advantages of analytical methods is that, not requiring time-consuming simulations, the effects of eventual parameters present in the model can be investigated in detail. An analytical form of FC has been proposed by Tononi, Sporns and Edelman [33] for a time discrete linear model in a study proposing a new measure for network complexity. An assumption behind this derivation has been later criticized by Barnett et al. [34], who proposed a new derivation of the FC of a linear stochastic model of the Ornstein-Uhlenbeck type providing a matrix operation to be applied over SC. Different derivations of this result have also been provided by Galàn et al. [35], by Deco et al. [14] through the use of Fokker-Planck equation applied to a linearized mean field model, by Robinson and colleagues [36] applying propagator methods. Here we propose another derivation of FC by making explicit use of the solution of Uhlenbeck and Ornstein model [37] and, most importantly, we go further in the analysis by inverting the solution: we find that SC can be inferred from functional data without the need of performing any parameter exploration.

At present, efforts are being made to retrieve SC from functional data, both in a model or data driven fashion. Model driven approaches are often restricted to a small number of regions [38], require iterative-fitting and optimization, as for example in [39], or, when analytical, results could not be extended or applied to all FC’s components [25, 40] or required parameter fitting [30]. Simpler data driven approaches based on partial correlation or regularized inverse covariance proved to be highly successful in retrieving structural information from various simulated time series [38, 41, 42], and have been applied to different brain functional data [43]. We demonstrate that the analytic SC of the linear model is equivalent to partial correlations, which are inversely proportional to inverse covariance. The link between inverse covariance and structure for a multivariate stochastic linear model is well known [44].

We first present the results for the analytically predicted FC (aFC), and, our main result, the analytically predicted SC (aSC). We compare the results with empirical data and with simulations obtained through a more biologically sophisticated model. We then take advantage of the application of the analytical operations, which facilitate ease of use and detailed parameter explorations.

## Results

We modeled large-scale resting state brain dynamics using simple linear diffusion of noise over the anatomical structure. This is the well known Ornstein-Uhlenbeck model as described for example in [35], where Galán applied this model to investigate micro-scale networks.

To test the results against empirical data, we used 20 minutes resting-state fMRI BOLD signal time series for 14 healthy subjects. For the same subjects we also had structural data from DTI and tractography. The parcellation used is a modified version of the Desikan-Killany atlas, as described in [16], which comprises 66 areas.

The details of the data, model and the analytic treatment can be found in Materials and Methods.

### Predicting FC from SC

#### Matrix operation

The analytic operation used to compute the covariance from SC is

$$\begin{array}{c} C=-\frac{\sigma^{2}}{2}{\left(-I+cW\right)}^{-1} \end{array}.$$ (1)

where **W** is equal to SC with the diagonal removed if present. The free parameters of the model are: *σ*, the standard deviation of the white Gaussian noise and *c*, the global coupling parameter accounting for the global strength of couplings in the system. In this formulation, SC is based upon a symmetric SC, as obtained from tractography. An extension to asymmetric SC is provided in Materials and Methods. PCC can be straightforwardly computed from the covariance matrix.

PCC is a common measure for stationary FC (as, for example, in [22]). The advantages over the use of covariance is that the entries are all normalized with regard to the variances, this makes comparisons among subjects and among studies easier. We will thus use it whenever possible.

We applied the algorithm to the mean SC of *N* = 14 subjects [45, 46] in order to predict the mean FC. We also exploited the low computational cost of the method to compute single subjects FC (based on single-subject SC) and average them to compare the two procedures, which may in fact be different given the non-linearities of the analytical operation.

#### Role of global coupling

**Fig 1:**
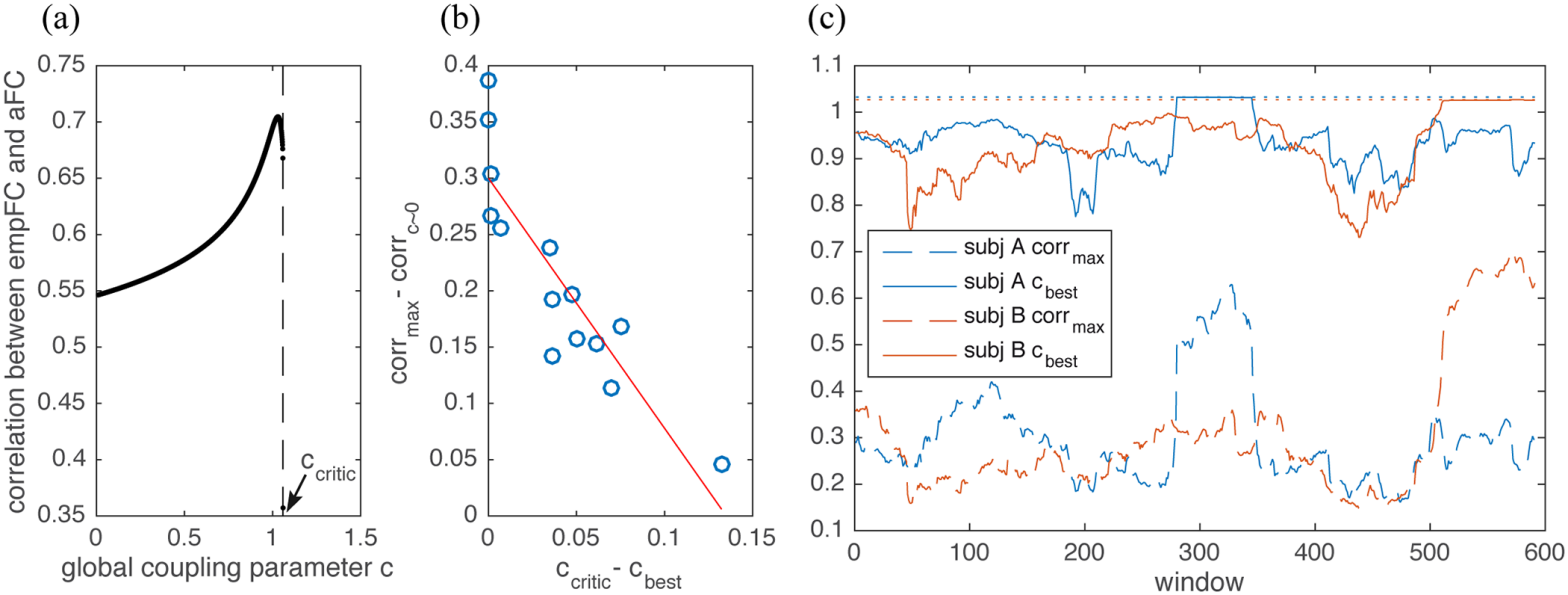
Role for global coupling parameter *c*. (a) Correlation between the empirical FC (mean across 14 subjects) and the linear analytical one (again, mean across the 14 subjects), for various values of the global coupling parameter c. The measure used for FC is Pearson Correlation Coefficients. (b) For each of the 14 subjects is shown the difference between the critical value of the parameter and the best fitting one versus the gain in correlation (the difference between the correlation obtained for the best fitting parameter and that for *c* ∼ 0). The closer the best value of the parameter to the critical value the higher the gain in correlation. (c) Results for the sliding-windows analysis for two subjects, labeled A and B: the analytical operator to obtain aFC has been applied to each window. Solid line shows the behavior of the best fitting parameter and the dashed one indicates the correlation reached between empirical and analytical FCs. Dotted lines indicate the value of *c*_*critic*_.

As seen from Eq (1), *c* is the only free parameter for FC. Noise strength, in fact, only appears as a scaling factor and will not affect Pearson correlation, which is the measure we used to compare the analytically predicted FC (aFC) and the empirical FC (empFC) from human imaging data. We demonstrate the big benefit of the analytic approach that renders computationally costly simulations unnecessary and allows for efficient explorations of the parameter space spanned by the coupling parameter c. The explored parameter’s range goes from *c* = 0, i.e. uncoupled nodes, to *c*_*critic*_ = 1/*max*_*i*_ *λ*_*i*_, where *λ*_*i*_ are the eigenvalues of **W**. The equilibrium point of the system loses stability at the latter value *c*_*critic*_. Correlations between the predicted aFC and the empFC for different parameter values are shown in Fig 1a.

**Fig 2:**
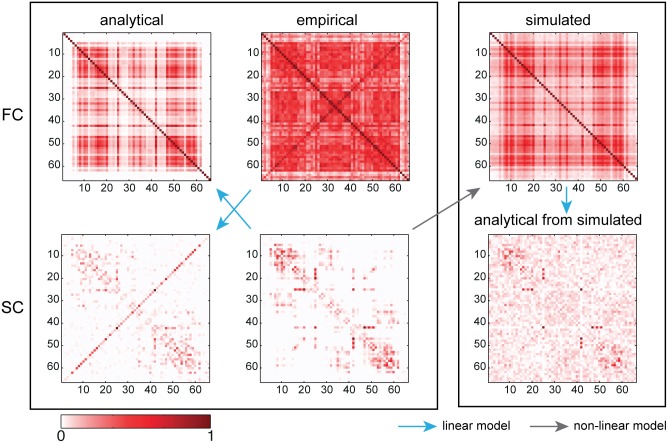
Structural and functional mean connectivities. Empirical mean empFC was obtained from resting-state fMRI BOLD time-series (top, middle) and used to generate the linear analytically inferred aSC (bottom, left). Structural data from DTI (bottom, middle) were the base to generate both the linear analytically predicted aFC (top, left) and to simulate time series through a model exhibiting non-linear dynamics to produce a simulated sFC (top, right). The latter has then been used to analytically infer again SC (bottom, right) to show how the non-linearities present in the model used to generate sFC do not prevent the linear retrieving of SC. The range of values in the FCs goes from zero to one. We used the same scale for the SCs (correlations between connectivities are independent from the scaling factor).

The absence of finite time simulation effects in our aFC allows to explore in details how the global coupling affects the diffusion of noise on structure (S1 Fig). When *c* equals zero, in fact, aFC will be an identity matrix as the values of noise injected in different nodes are independent and diffusion is precluded. For small couplings, the noise is enabled to diffuse to adjacent nodes. This leads to a correlation, if the diagonals are neglected, which at first order (see Materials and Methods for a mathematical argument) reflects the similarity between empSC and the empirical Cov (empCov), against the zero correlation observed when simulating data for the same model [26]. The difference to computationally simulated data is that these emergent functional correlations are several orders of magnitude smaller than the variance of each node and are thus masked by noise if the simulated time-series are not long enough. Further increasing the strength of the coupling, functional correlations appear between structurally unconnected regions and their weights increase with *c*. The maximum of the correlation for the averaging before method is 0.54 (*CI* = 0.45−0.57), reached for *c*_*best*_ = 1.03, which is close to the boundary between stability and instability (the critical value is *c*_*critic*_ = 1.06) as shown in Fig 1a. The maximum of correlation for the averaging in the end procedure is instead 0.64 (*CI* = 0.59−0.67), with a different value of *c*_*best*_ (in all cases in the proximity of *c*_*critic*_) for each subject. The correlation is computed as Pearson correlation between the matrices’ lower triangles, diagonal excluded. We find it useful, though, to maintain the diagonals when performing the parameter exploration as this allows to obtain a better scaling between variances and covariances. This explains the higher correlations which appear in Fig 1a. Once that the best value of the parameter has been identified, we removed the diagonals and computed the correlation again to avoid the positive bias. The best analytically predicted FC (with averaging towards the end) is shown in Fig 2 (top, left), together with the empirical one (top, middle).

In Fig 1b we plot, for each subject, the gain in correlation between analytical and empirical data reached during parameter exploration (i.e. the difference between the correlation obtained for *c*_*best*_ and that for *c* ∼ 0) versus the distance of *c*_*best*_ from the critical value *c*_*critic*_. The importance of being close to criticality has been stressed by different authors (see for example [16]) and we here find that the closest the best fitting value is to to the critical one, the bigger is the gain in correlation.

#### Comparison with a non-linear model

To corroborate the results of [22] on the debate linear versus nonlinear models, we chose a more biophysically realistic non-linear model as described in [14], which comprises spiking neurons and AMPA, NMDA, and GABA synapses. Simulations of the network show that the simulated FC (sFC) converges to the aFC. For simulated neural activity of 20min duration (1200000 simulated points) the correlation between sFC and aFC is 0.95 *CI* = 0.94−0.96 (see Material and Methods for further details). The sFC is shown in Fig 2 (top, right).

### Inferring SC from FC

#### Matrix operation

To infer an improved approximation of SC from functional data, we invert the analytical operation in Eq (1):

$$\begin{array}{c} W=-\frac{\sigma^{2}}{2c}C^{-1} \end{array}$$ (2)

The diagonal is set to zero to avoid offsets. The procedure is based on the same assumptions, but in this case no extension to asymmetrical SC is possible. For this inverse operation, both free parameters of the model appear as scaling factors and will not affect the correlation with the empirical SC, so no parameter exploration is needed. Note that this time the operation requires the use of the covariance as a measure of functional connectivity.

#### Predicting structural connectivity

We applied the algorithm, to infer a mean analytical SC, both to the mean empCov and to single subjects empCovs, which were then averaged. At the single subject level, the mean correlation between empSC and aSC was 0.46 with a standard deviation (SD) of 0.02. For the averaged data, correlation was 0.57 (*CI* = 0.46−0.70) when averaging single subjects aSCs and 0.53 (*CI* = 0.42−0.67) when the analytical operation was applied directly to the mean empCov. The best predicted aSC (obtained averaging at the end) is shown in Fig 2 (bottom, left). The aSC obtained displayed small negative entries, possibly due to noise in the empirical functional data. We removed these negative entries, which have no meaning as structural connections, without applying any further thresholding. For the aSC shown in Fig 2, the mean and std of the negative connections were −0.02 and 0.02 respectively.

#### Resolving homotopic connections

The most striking difference between SC produced by diffusion imaging and the analytical SC (aSC) derived analytically from functional data, is that the latter features strong homotopic connections (they can be observed on the antidiagonal), which are weaker in the former. Tractography, as shown in [47], may lead to an underestimation of the strength of these inter-hemispheric connections.

When correlations between empirical data and analytical results are computed separately for the intra and inter hemispheric connections, they result respectively in correlations of 0.68 and 0.62 (S2 Fig).

#### Impact of the non-linearities of a biophysical model

Our analytic operation provides aSC from empCov and is independent of both the global coupling parameter and the noise variance. We applied the inverse algorithm to the sCov previously simulated through the non-linear model (see Materials and Methods), and obtained an analytic SC (aSC-nl), which resembled the original SC used to generate the simulated data (0.73 of correlation, *CI* = 0.63−0.83, using the 20min time-series). aSC-nl is shown in Fig 2 (bottom, left). We computed also sCov for larger values of the noise, 0.001 rather than 0.00005, and applied the inverse operation. The correlation with empSC is 0.82 (*CI* = 0.71−0.87) for same simulation length, i.e. higher than before, and increased to 0.92 (*CI* = 0.88−0.95) for 1h simulation.

### Inter and intra-subject variability

#### Variability and data reliability

The question arises whether it is possible to predict subject specific connectivities through the analytical operators. Therefore, we first quantified the variability among single subject’s data. We computed Pearson correlations between subjects’ SCs, and between subjects’ FCs. SCs were found to be more similar between each pair of subjects (correlation mean of 0.82 and SD of 0.07) than the FCs (correlation mean 0.6 and SD 0.07).

**Fig 3:**
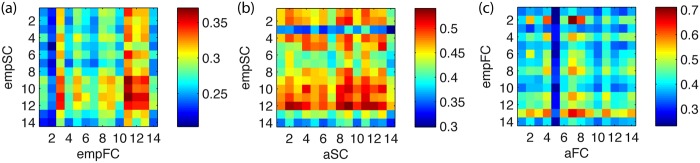
Across subjects variability. In the three matrices we show correlation between connectivities of the 14 subjects: (a) correlation between empSC and empFC; (b) correlation between empFC and aFC (derived from empSC); (c) correlation between empSC and aSC (derived from empFC) in the right panel. It is not possible to link subject specific empirical connectivities and variability remains between the individual analytic and empirical connectivities.

In Fig 3a we show the correlation between empSC and empFC for each pair of subjects. However, despite the variability in the empirical data, subject specific connectivieties cannot be linked.

Furthermore, variability remains between the individual analytic and empirical connectivities, as shown in Fig 3b and 3c.

#### Analytical operations for sliding-windows analysis

Due to their analytical nature, the operations so far proposed can be applied to sliding-windows at a low computational cost to explore the temporal evolution of the time-series. As a proof of concept we performed two analyses, one with the analytical operation for FC and one with that for SC. For both analyses we used a window size of 140s and a step size of 2s. The window size has been chosen big enough to avoid numerical errors when performing the matrix inversion required to obtain aSC.

We first computed the empirical FC for each window, empFC_w_, and then applied the analytical operation to empSC in order to obtain aFC_w_. We could thus investigate how the global coupling parameter *c* varies through the resting-state scan session. Results for two subjects, labeled A and B, are reported in Fig 1c. Again, we can observe how proximity to criticality positively affects the agreement between analytical and empirical data.

In the second analysis, we applied to the analytical operation to empCov_w_ in order to obtain aSC_w_. This allows to highlight, for each window, which are the anatomical links that are involved in generating functional connectivity. For each link we also computed the standard deviation across windows. A small standard deviation signifies a stable contribution to function, a fluctuating contribution instead results in big standard deviation. Results are shown in S3 Fig.

#### Time-dependent coefficients for the projectors

**Fig 4:**
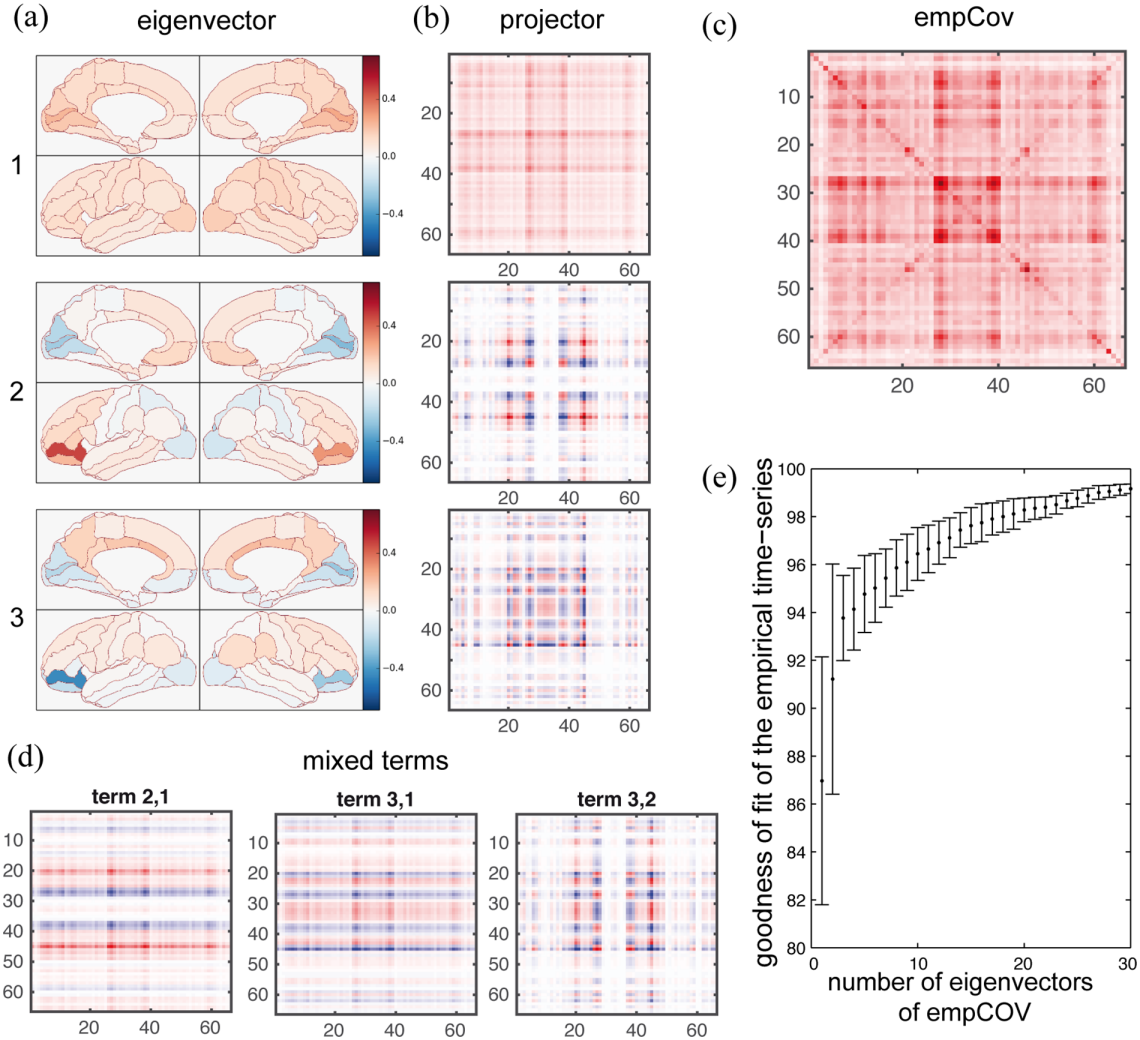
Eigenvectors, projectors and dimension reduction. Two visualization tools are given in this figure. (a) The eigenvectors of the empCov (which, in our model, are the same as aSC) are plotted on the brain surface in arbitrary units. (b) The portion of empCov (or aSC), which is strictly dependent on each eigenvector is pictured at its side through the use of projectors. (c) The average empCov. (d) The mixed terms relative to the first three eigenvectors. (e) For each single subject, we computed the goodness of fit between the complete time-series (spanning a 66 dimensional space) and a version of them projected on a smaller subspace composed of only a limited amount of the empCov eigenvectors. For each number of eigenvectors considered, we plotted the mean goodness of fit and the standard deviation across subjects. It appears that a three dimensional subspace is enough to account for almost 94% of the data.

In Material and Methods we describe how projectors can be computed for Cov. Projectors express the portion of the matrix that is strictly pertinent to each eigenvector and can be used as an intuitive visualization tool, as shown in Fig 4a and 4b. In particular, the linear model predicts that Cov and SC have the same eigenvectors, and thus the same projectors. Any symmetric matrix, as the ones we are dealing with, can be written as a linear combination of its eigenvectors’s projectors, where the coefficient of each is the relative eigenvalue. This implies that both Cov and SC can be written as a linear combination of the same *n* projectors matrices (while a basis for a *n* x *n* symmetric matrix generally requires *n*(*n* + 1)/2 elements). The difference between Cov and SC, in this model, is given only by the difference in the weights of these projectors. In addition, the weights of Cov are a function of the weights of SC.

**Fig 5:**
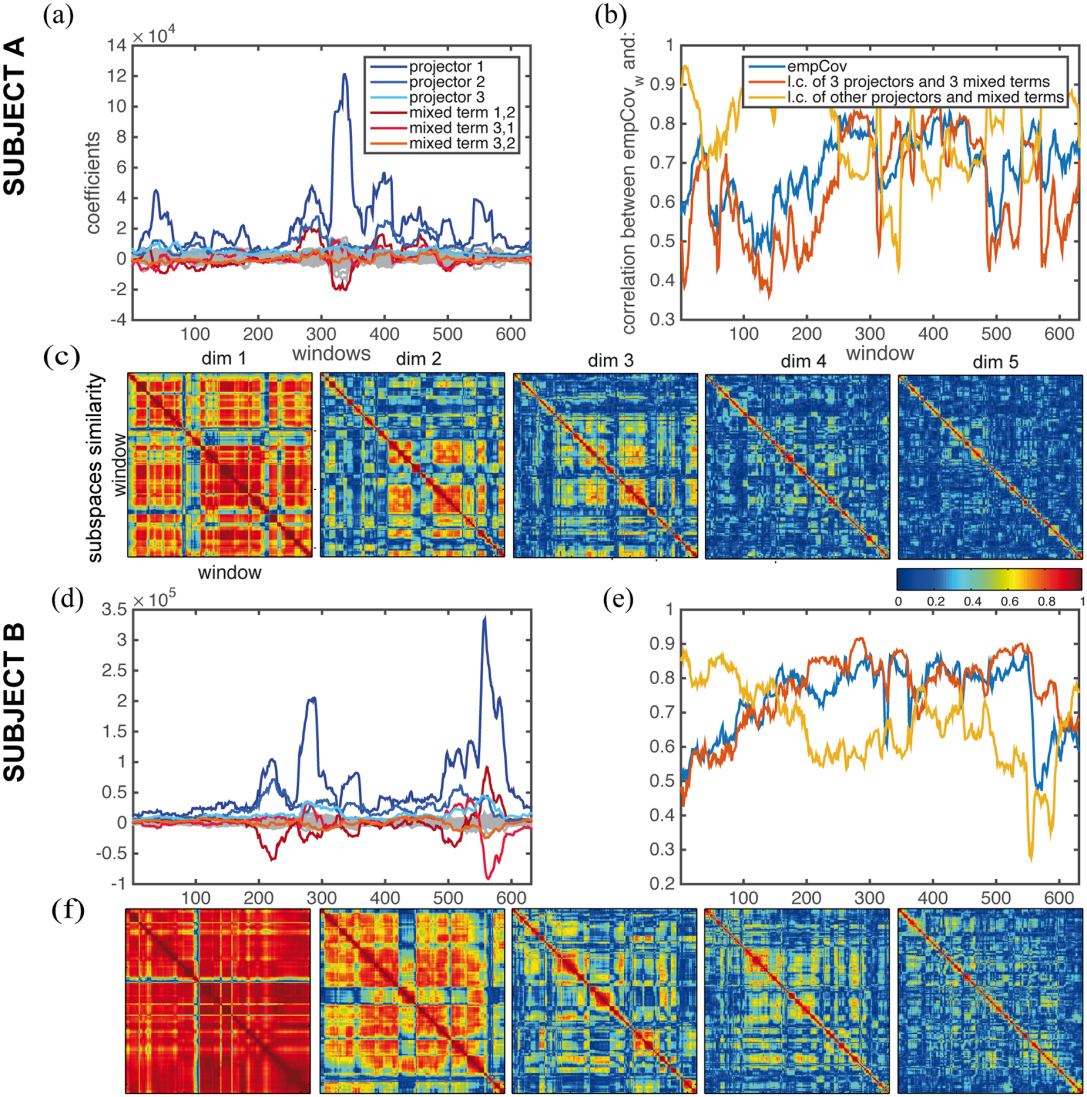
Decomposition of resting-state fluctuations. We performed single subjects sliding-window analysis (window size 60s, step 2s) of the empirical data and in this set of figures we show the results for the two subjects A and B. The total empCov can be written as a linear combination of its 66 projectors each strictly linked to one of the eigenvectors. Each window empCov_w_ can then be decomposed in a linear combination of projectors of empCov and a residual part accounting for mixed eigenvectors terms. (a,d) Time-evolution of the coefficients used in the linear combination of projectors and mixed terms appears to strongly fluctuate in time. A small number of terms (the first three projectors and three mixed terms) have a higher weight in the linear combination than the others, and also seem to exhibit a higher variability in time. (b,e) The correlation between each window’s covariance and the linear combination of the first three projectors and three mixed terms is plotted, together with the correlation of the window covariance with the linear combination of the other projectors and mixed terms. We also show the correlation between the window covariance and the total covariance (c,f) For each window we computed the window’s Cov and its eigenvectors. We ranked the eigenvectors according to the value of their eigenvalues and selected up to five of them as a basis for a subspace of the measure’s space. We built subspaces of dimension one through five (matrices from left to right). For each dimension, we show a matrix of which the entries represent a measure of similarity between the subspaces of different windows. The canonical correlatioin is used as a measure of similarity, where 1 means that the subspaces are identical and 0 that there is no similarity.

To explore whether the non-stationarities inherently present in the correlations of the empirical data [27] could be nonetheless decomposed in a meaningful way as a linear combination of the same projectors of the total empCov, we performed a sliding-window analysis (window size 60s, step size 2s, as suggested in [48]). This follows the spirit of Galán and colleagues [35] who investigated how timeseries can be decomposed in terms of their eigenvectors, with the difference that we focus on the decomposition of the time varying covariance matrix. The modulation in time of the weights of each of the 66 projectors is shown in Fig 5a and 5d for two subjects labeled A and B. In the figure the coefficients of the three projectors derived from the principal eigenvectors are shown in blue, the others in grey. Using these coefficients, we built a time-dependent linear combination of projectors, and correlated it with the window’s covariance (empCov_w_). Residuals, or mixed terms, which take into account the terms other than the projectors (see Materials and Methods), were also computed and correlated window by window with the window’s covariance. After averaging across windows and across subjects, linear combinations of all the projectors resulted in a correlation of 0.79 with an average standard deviation (SD) of.07, but the residual terms also played an important role with a correlation of 0.59 and an average SD of 0.12. In figure Fig 5a and 5d we also show the coefficients of the mixed terms: the mixed terms relative to the first three eigenvectors are shown in shades of red, the others in grey.

#### Dimension reduction

The first three projectors and three mixed terms give the strongest contribution to the linear combination, see Fig 5a and 5d. To illustrate the effects of reduction of the dimensionality in terms of loss of information as expressed in the time series, we computed the goodness of fit of the lower dimensional data as explained in [49] (Fig 4c). The three most important eigenvectors (i.e. relative to the highest eigenvalues) gave a goodness of fi of around 94%. The time-dependent linear combination of the three projectors and three related mixed terms can be used to build a time varying reduced empCov_w_. This reduced covariance matrix yields an average correlation (across time and subjects) of 0.74 with the full empCov_w_ (correlations are shown in Fig 5b and 5e for the two subjects). This reflects the average correlation, 0.75, between the total covariance and the covariance of the window. Using six matrices to build the Cov is more restrictive than using three eigenvectors for the time series. In fact, the dimension of the state time series space is *n* = 66, while a base for the Cov space would require *n*(*n* + 1)/2 = 2211 matrices. Considering the linear combination of the remaining 2205 matrices yielded an average correlation of 0.65 with empCov_w_.

We then considered how the most important eigenvectors evolved in time. For each subject we performed again a sliding-window analysis. For each window empCov_w_ was computed together with its eigenvectors. The eigenvectors have been ranked according to the corresponding eigenvalue and the first five eigenvectors have been used to build subspaces of dimension from one to five. Fig 5c and 5f illustrates the degree of intra-subject variability of the computed subspaces in which empCov evolves. The similarity between pairs of subspaces (measured as the cosine of the angle between the subspaces) is around 0.88 for one dimension and decreases to 0.18 for higher dimensions. As for the SD, it slightly increases (from.10 for the first dimension to.16 for the fifth).

## Discussion

We have here derived a set of analytical methods that offers useful approximations both in predicting long-term simulated data and in analyzing empirical data, suited for example to perform fast preliminary explorations or for more efficient studies of within and between subjects variability. Given the analytic nature of our approach, the analysis is efficient as it avoids the time-consuming simulations that are usually required by both linear and non-linear models. By construction, the details on the temporal evolution are not provided by the analytically derived connectivity and operations, which, in fact, are not based on time-series simulations. Nevertheless, some insights on the evolution of the spatiotemporal organization of real data can be gained through our approach by applying the analytical operations to sliding-windows.

We propose an inverse analytical operation to retrieve SC from functional data. Previous approaches have derived SC either by simple thresholding of FC (for example, [2]) or by an inversion limited to FC’s main component (see [40]). Robinson et al. [40] also provided analytical results for a linear model similar to the one used in the present work. An identical model, instead, has been used by Dominguez et al. [30] to derive an analytical expression for the SC, starting from the time-lagged covariance. The dependence from the time-lag is present in the analytical expression for SC and thus requires parameter optimisation. The advantage of the present approach is that it is parameter free. This is also an advantage when comparing the present approach with a recent computational approach proposed by Deco et al. [39]. In their work the authors used dynamic mean-field network models to derive a new SC matrix from the FC matrix using an iterative-fitting and optimization algorithm [39]. The authors found that a good improvement in the correlation between empirical and simulated FC matrices was obtained with the addition of a small number of anatomical links to the empirical SC and reweighting of existing connections. The addition of these links allowed the authors to improve the fitting between empFC and the sFC simulated from the improved SC up to a correlation of 0.75. In the present work, the analytical FC obtained by applying again the analytical operation to aSC is empFC itself, thus this correlation would be 1. It appears that the necessary extra links found by Deco et al. are of the same nature of those retrieved in the present work, particularly cross-hemispheric homotopic connections. However, the computational procedure required by Deco et al. was time consuming and relied on the hypothesis that the structure-function relation is maximal when the global network dynamics operates at the critical point of instability of the equilibrium state. The criticality hypothesis relates particularly well to the stationary components of the FC, but not the non-stationary switches between epochs of invariant FC as found by [26]. In the present work, we did not rely on a hypothesis related to criticality. In fact the present approach is completely agnostic regarding the coupling strength, since it appears only as a scaling parameter of the inverse operation. The here proposed analytical operation, though, is only valid for symmetrical SCs, such as those obtained from tractography. In case of asymmetries, there are infinite solutions to the inverse problem and different strategies should be employed to approximate SC. An iterative minimization procedure which could be used to address this problem is described, for example, by Steinke and colleagues [50].

The analytical aSC derived from functional data correlates around 0.6 with the empirical SC derived from tractographic reconstruction, empSC. In the present work it was not possible to link subject specific structural and functional connectivities, nor empirical and analytical ones. This could be due to the homogeneous quality of our sample, healthy subjects of similar age, but could also be a consequence of limitations in the measures. For example, Anderson and colleagues showed that at least 25 minutes of rs-fMRI BOLD registration are required in order to reliably discriminate a single subject’s FC from the population [51]. It would thus be interesting to explore whether the use of longer time-series, which represent a better sampling of the resting-state activity, would allow to obtain an improved estimate of SC at the individual level. There are also principal considerations that prevent us from attempting further quantitative improvements of this correlation. First, tractography has its own limitations, for example its capacity to reconstruct inter-hemispherical or long-range weak connections [47, 52] is limited. Thus, a first limit in the correlation between empirical and analytical data is the nature of the empirical data itself. The prominent role played by these underestimated homotopic connections in shaping FC has already been highlighted by Messé et al. [22] and by Deco et al. [39] and is evident also in our work. In fact, when restricting the correlation to only intra-hemispherical connections, the correlation reaches 0.68. Second, the present approach assumes that resting-state activity is stationary which has been shown not to be the case [26, 27]. Nevertheless, the stationary FC contains relevant information characterizing the network dynamics and aSC may serve as a starting point for iterative methods extendable to other models, such as in Deco et al. [39].

It has been shown that data-driven approaches based on correlations such as partial correlation or regularized inverse covariance can provide a good estimate of SC when applied to functional data [38, 41, 42]. A systematic investigation of the power of these and other methods has been performed by [38]. The authors generated simulated data for different conditions, using different models and adding various problematic confounding elements. They then tested various methods for assessing the effective connectivity of the network. When connection directionality was neglected, partial correlation, regularized inverse covariance, and several Bayes net methods outperformed the other ones. When non-stationary data, or directionality of connections were considered, the first two methods lost predictive power. For the linear model used in this work partial correlation corresponds to structure and is inversely proportional to the inverse covariance. These linear approaches, though, have not always provided the best estimate for anatomical connections. Watanabe and collegues [53] have shown that a model that is not relying on the assumption of stationarity, the pairwise maximum entropy model, outperformed partial correlation in two of the resting state networks. It would be interesting to extend the study to the whole brain. A better characterization of resting-state non-stationarities would improve our understanding of how function arises from the underlying structure.

Overall, the simplicity of the analytical operations here proposed permits rapid routine use in first approximations of interhemispheric connections in DTI-derived SC to possibly complement or guide tractography, or could provide information when structural data are missing and only functional data are available. In addition, it is useful to provide a method for retrieving SC or FC from a different kind of data than those typically used, as this can help to disentangle the effect of systematic errors in the type of measurement. It would also be interesting to see whether aSC could be used to investigate alterations in the connectivity in diseases, like Asperger’s syndrome [30]. Potentially the analytical results can serve as a biomarker for diseases that alter brain connectivity.

## Materials and Methods

### Data collection

Structural data from DTI and resting-state BOLD signal time series were acquired for 14 healthy subjects (age between 25 and 33 years old, 6 females). Data can be found in S1 Folder.

A detailed description of the generation of SC and FC matrices from those data can be found in [46].

Empirical data were acquired at Berlin Center for Advanced Imaging, Charité University Medicine, Berlin, Germany. All participants of this study gave written informed consent before the study, which was performed in compliance with the relevant laws and institutional guidelines and approved by the ethics committee of the Charité University Berlin. For functional imaging (EEG-fMRI) [54, 55], subjects were asked to keep awake and keep their eyes closed—no other controlled task had to be performed. In addition a localizer, DTI and T2 sequence were recorded for each subject. MRI was performed using a 3 Tesla Siemens Trim Trio MR scanner and a 12-channel Siemens head coil. Specifications for the employed sequences can be found in [46]. Each scan session started a localizer sequence (TR 20 ms, TE 5 ms, 3 slices (8 mm), voxel size 1.9 × 1.5 × 8.0 mm, FA 40°, FoV 280 mm, 192x192 matrix). For each participant anatomical T1-weighted scans (TR 1900 ms, TE 2.25 ms, 192 sagittal slices (1.0 mm), voxel size 1 × 1 × 1 mm, FA 9°, FoV 256 mm, 256x256 matrix) as well as T2-weighted scans (TR 2640 ms, TE1 11 ms, TE2 89 ms, 48 slices (3.0 mm), voxel size 0.9 × 0.9 × 3 mm, FA 150°, FoV 220 mm, 256x256 matrix) were acquired. Diffusion-Tensor-Imaging (TR 7500 ms, TE 86 ms, 61 transversal slices (2.0 mm), voxel size 2.3 × 2.3 × 2.3 mm, FoV 220 mm, 96x96 matrix) and GRE field mapping (TR 674 ms, TE1 5.09 ms, TE2 7.55 ms, 61 transversal slices (2.3 mm), voxel size 2.3 × 2.3 × 2.3 mm, FA 60°, FoV 220 mm, 96x96 matrix) were measured directly after the anatomical scans. After dw-MRI acquisition, the subject was moved outside the scanner room, the EEG-setup was prepared and the subject was placed inside the scanner again. After another localizer sequence functional MRI (BOLD-sensitive, T2*-weighted, TR 1940 ms, TE 30 ms, FA 78°, 32 transversal slices (3 mm), voxel size 3 × 3 × 3 mm, FoV 192 mm, 64x64 matrix was recorded simultaneously to the EEG recording.

Processing steps executed by the Berlin automatized processing pipeline [46]:

1. Preprocessing of T1-weighted scans, cortical reconstruction, tessellation and parcellation
2. Transformation of anatomical masks to diffusion space
3. Processing of diffusion data
4. Transformation of anatomical masks to fMRI space
5. Processing of fMRI data

#### Parcellation

The highly resolved anatomical images are important to create a precise parcellation of the brain. The parcellation is required for different purposes. When computing fiber tracts, the gray matter / white matter (GM/WM) segmentation is used as mask to ensure that fiber tracking starts at the GM-WM matter border and that tracks are restricted to the WM. Parcellation is also important for splitting the GM in anatomically informed functional units or regions. For each of those parcellated units, empirical functional data time series are spatially aggregated.

T1-weighted images are pre-processed using FREESURFER’s recon_all function. Pre-processing includes the following processing steps:

1. Motion correction, intensity normalization/correction
2. Skull stripping, removal of all non-brain tissue, brain mask generation (brainmask.mgz).
3. WM and subcortical segmentation, cortical tessellation generating GM-WM and GM-pia interface surface-triangulations, probabilistic atlas based cortical parcellation, e.g., using Desikan-Killany (DK) atlas [56]. This generates aparc+aseg.mgz volumes that contain all parcellated regions (cortical and subcortical) with corresponding region labels used for fiber-tracking and BOLD time-series extraction. Diffusion-weighted MRI (dwMRI) data are parcellated according to the high-resolution atlases derived from T1-weighted data yielding estimated white matter fiber tracts and SC matrices. The parcellations are used for defining seed- and stop-locations during tractography.

#### Tractography

Tractography requires binary WM masks to restrict tracking to WM voxels. WM masks ensure that none of the tracks extend into GM. Using FSL’s fslmath function GM is removed from the segmentation mask aparc+aseg2diff.nii. Since MRTrix and other tracking toolboxes enable sub-voxel tracking, tracking masks are created from FREESURFER’s high-resolution parcellations. Next, WM mask volume and WM outline volume are merged and a binary mask volume is obtained.

Upon extraction of gradient vectors and values (known as b-table) using MRTrix, dw-MRI data are pre-processed using FREESURFER’s Using the registration rule created by FREESURFER’s function dt_recon we transform the high-resolution mask volumes from the anatomical space to the subject’s diffusion space, which will be used for fiber tracking. The cortical and subcortical parcellations contained in aparc+aseg.nii are resampled into diffusion space, one time using the original 1 mm isotropic voxel size (for subvoxel seeding) and one time matching that of our dw-MRI data, i.e., 2.3 mm isotropic voxel size.

During MRTrix pre-processing diffusion tensor images that store the diffusion tensor (i.e., the diffusion ellipsoid) for each voxel location are computed. Based on that, a fractional anisotropy (FA) and an eigenvector map are computed and masked by the binary WM mask created previously. For subsequent fiber-response function estimation, a mask containing high-anisotropy voxels is computed. In order to resolve crossing pathways, fibers are prolonged by employing a probabilistic tracking approach as provided by MRTrix. It is based on a constrained spherical deconvolution (CSD) that computes the fODF for each image voxel [57, 58].

In order to exclude spurious tracks, three types of masks are used to constrain tracking: seeding-, target- and stop-masks. In order to restrict track-prolongation to WM, a WM-mask that contains the union of GM-WM-interface and cortical WM voxels is defined as a global stop mask for tracking (MRTrix streamtrack command option ‘-mask’). To address several confounds in the estimation of connection strengths (information transmission capacities), a new seeding and fiber aggregation strategy was developed for this pipeline. In combination with a new aggregation scheme, it is based on an appropriate selection of seed voxels and controlling for the number of generated tracks in each seed voxel. Instead of using every white matter voxel, tracks are initiated from GM-WM-interface voxels and a fixed number of tracks are generated for each seed-voxel. Since a GM parcellation-based aggregation is performed, each seed-mask is associated with a ROI of the GM atlas. Along with seeding-masks complementary target-masks are defined specifying valid terminal regions for each track that was initiated in a specific seed voxel.

The capacity measures that we derive between each pair of regions are intended to estimate the strength of the influence that one region exerts over another, i.e., their SC. A serious confound arises from the dependency of the number of found tracks on path-length and on the shape of the diffusion orientation profile along the pathway due to streamline dispersivity [59]. Due to step-wise dispersion of the propagating streamline the probability that a specific track is prolonged decreases as a function of the distance from the seed point. This creates a bias towards short pathways and pathways that follow the major diffusion directions. In order to improve existing methods for capacities estimation and to address the aforementioned confounds, we make use of several assumptions with regard to seed-ROI selection, tracking and aggregation of generated tracks [46].

Upon tractography the pipeline aggregates generated tracks to structural connectome matrices and outputs three metrics that quantify the capacities of individual whole-brain connectivity.

- Raw counts: track counts of all tracks that were found between each pair of regions (yielding symmetric capacities matrix).
- Distinct connection counts: only distinct connections between each pair of regions (yielding symmetric capacities matrix). This has been used in the present work.
- Weighted distinct connection counts: each distinct connection is divided by the number of distinct connections leaving the seed-voxel (yielding asymmetric capacities matrix).

Each of these metrics are outputted as two variants, namely, absolute values and relative values that have been normalized by the total surface area of the GWI of a subject.

#### FMRI data processing

In order to generate the functional connectivity (FC) matrices, raw fMRI DICOM files are first converted into a single 4D Nifti image file. After this step, FSL’s FEAT pipeline is used to perform the following operations: deleting the first five images of the series to exclude possible saturation effects in the images, high-pass temporal filtering (100 seconds high-pass filter), motion correction, brain extraction and a 6 DOF linear registration to the MNI space.

Functional data is registered to the subject’s T1-weighted images and parcellated according to FREESURFER’s cortical segmentation. By inverting the mapping rule found by registration, anatomical segmentations are mapped onto the functional space. Finally, average BOLD signal time series for each region are generated by computing the mean over all voxel time-series of each region. From the region wise aggregated BOLD data, FC matrices are computed within MATLAB using pairwise mutual information (on z-transformed data), and Pearson’s linear correlation coefficient as FC metrics.

Any pre-processing technique, which implies a normalization of data must be avoided when using our analytic operation, for this reason it is important to stress that we did not perform global signal regression on data. Global regression, in fact, changes the distribution of the eigenvalues of the FC and, in particular, shifts the correlations towards negative values. In resting-state BOLD data, this means that zero and negative correlations are introduced. While the debate on the meaning of these negative correlations and on the appropriateness of the use of global regression is open (see for example [60]), this procedure must absolutely be avoided when using the analytical operation here presented as the introduction of zero eigenvalues leads to the impossibility of inverting FC to obtain SC. Authors who worked with similar analytical operations, have in fact found difficulties in dealing with regressed data [40], and addressed the problem by removing all the non-positive eigenvalues before applying the operation. This, not only causes the loss of some information, but it is also based on the assumption that the distribution of the positive eigenvalues is unaffected by global regression, which is not the case as all the eigenvalues become more negative.

In the present work we used the 66 regions parcellation presented in [16], and computed FCs for each subject using either Pearson linear correlation coefficients (PCC) or computing covariances (Cov). PCC is the measure mainly used when talking about FC, its advantage over Cov is that the entries are normalized to the values of the variances and this allows, among other advantages, an easier comparison among subjects and different scan sessions. For this reason we presented results for PCC whenever possible. We also calculated mean SCs and FCs by averaging data across subjects.

### Analytical treatment

#### System and general solution

An activity variable *x*_*i*_(*t*) is assigned to each node in the network. The variable represents the activity of the i-th node at time *t*. A system of *n* regions is described by a vector variable **x**(*t*). The time evolution of the activity of each node depends on the activity of the other nodes, through a coupling matrix **A**, and on the additive Gaussian white noise. We are thus dealing with a linear system of *n* stochastic first order differential equations:

$$\begin{array}{c} \dot{x}\left(t\right)=Ax\left(t\right)+\sigma \xi \left(t\right) \end{array}$$ (3)

where *ξ* is Gaussian white noise and *σ* is the standard deviation.

In the matrix **A** we can separate the part relative to the self-connections, with weight −1, from the couplings between different nodes, which contain the weights as derived from the SC matrix multiplied by a positive *global coupling parameter* *c*:

$$\begin{array}{c} A=-I+cW \end{array}$$ (4)

The ‘minus’ sign for the self-connections guarantees that the nodes have a stable equilibrium point.

We use only one free parameter in the model, rather than one multiplying the identity and one **W** (as done in [25]), because the linear correlation measures chosen are insensitive to scaling factors, and we retrieve Eq (4) by dividing both sides of the equation of the two parameters model by the extra parameter.

In the present work we are comparing empirical BOLD signal activity with the activity simulated through a linear model. This means that this single parameter of the linear model does not have a predefined biological meaning but accounts for all the elements leading to BOLD signal, from neural activity to neuro-vascular coupling.

The model illustrated is well-known in the literature as the multidimensional Ornstein-Uhlenbeck process and the general solution, as presented for example in [61] is:

$$\begin{array}{c} x\left(t\right)=e^{tA}x_{0}+\sigma \int_{0}^{t}e^{(t-s)A}\xi \left(s\right)\;ds \end{array}$$ (5)

If all the eigenvalues of **A** are negative, which is the case for all positive values of *c* smaller than *c*_*critic*_ = 1/*max*_*i*_ *λ*_*i*_ where *λ*_*i*_ are the eigenvalues of **W**, then the first part of the solution will be a transient decaying to zero and the system will settle in its equilibrium state governed by the second part of the solution.

#### Covariance for the stationary state

In the stationary state, the covariance matrix between different components of the system can be evaluated for large *T* as follows:

$$\begin{array}{c} C=\frac{1}{T}\int_{0}^{T}x\left(t\right)x^{t}\left(t\right)\;dt \end{array}$$ (6)

where the super script *t* identifies the transpose.

Considering only the stationary part of the solution we obtain

$$\begin{array}{c} \begin{array}{cc} C & =\frac{1}{T}\int_{0}^{T}\left(\int_{0}^{t}e^{(t-s)A}\xi \left(s\right)\;ds\right)\left(\int_{0}^{t}e^{\left(t-r\right)A^{t}}\xi \left(r\right)\;dr\right)\;dt\\ & =\frac{\sigma^{2}}{T}\int_{0}^{T}\int_{0}^{t}e^{(t-s)A}e^{\left(t-s\right)A^{t}}\;ds\;dt \end{array} \end{array}$$ (7)

Where we have applied properties of Ito integrals. To multiply the two exponentials of matrices we need to apply Baker-Campbell-Hausdorff formula (truncating it at a sufficient order of approximation):

$$\begin{array}{c} e^{A}e^{A^{t}}=e^{Z} \end{array}$$ (8)

$$\begin{array}{c} Z=A+A^{t}+\left[A,A^{t}\right]+\frac{1}{2}\left(\left[A,\left[A,A^{t}\right]\right]-\left[A^{t},\left[A,A^{t}\right]\right]\right)+... \end{array}$$ (9)

so that the covariance takes the form

$$\begin{array}{c} C=\frac{\sigma^{2}}{T}\int_{0}^{T}\int_{0}^{t}e^{(t-s)Z}\;ds\;dt \end{array}$$ (10)

If **L** is the matrix containing the eigenvectors of **Z** and **Z**^**D**^ is the diagonalized form for **Z**, we can solve the integrals to obtain:

$$\begin{array}{c} C=L\left(-\sigma^{2}{\left(Z^{D}\right)}^{-1}\left(1-{\left(Z^{D}\right)}^{-1}\frac{\left(e^{TZ^{D}}-1\right)}{T}\right)\right)L^{-1} \end{array}$$ (11)

Taking into account that **Z** has negative eigenvalues, for T towards infinity we obtain:

$$\begin{array}{c} C=L\left(-\sigma^{2}{\left(Z^{D}\right)}^{-1}\right)L^{-1} \end{array}$$ (12)

in the case of symmetrical **A** we have that **Z**^**D**^ = 2**A**^**D**^ and the covariance reduces to:

$$\begin{array}{c} C=L\left(-\frac{\sigma^{2}{\left(A^{D}\right)}^{-1}}{2}\right)L^{-1} \end{array}$$ (13)

$$\begin{array}{c} C=-\frac{\sigma^{2}}{2}A^{-1}. \end{array}$$ (14)

If *σ* is not uniform, then *σ*^2^ is replaced by **Σ** = σ σ^**t**^. The Pearson correlation coefficients, and thus FC, can be computed by

$$\begin{array}{c} R_{ij}=\frac{C_{ij}}{\sqrt{C_{ii}C_{jj}}} \end{array}$$ (15)

#### Obtaining SC from the covariance

Eq (14) can be inverted to provide

$$\begin{array}{c} A=L\left(-\frac{\sigma^{2}}{2}{\left(C^{D}\right)}^{-1}\right)L^{-1} \end{array}$$ (16)

which, for **W**, gives:

$$\begin{array}{c} W=\frac{1}{c}L\left(I-\frac{\sigma^{2}}{2}{\left(C^{D}\right)}^{-1}\right)L^{-1}. \end{array}$$ (17)

$$\begin{array}{c} W=\frac{1}{c}\left(I-\frac{\sigma^{2}}{2}C^{-1}\right). \end{array}$$ (18)

As we are not interested in the diagonal, this can be removed and the inverse analytical operation simply becomes

$$\begin{array}{c} W_{i\neq j}=-\frac{\sigma^{2}}{2c}C_{i\neq j}^{-1} \end{array}$$ (19)

where both parameters of the model now appear as scaling factors.

Structure is thus inversely proportional to the inverse covariance.

When the covariance matrix *C* is invertible, the partial correlation coefficients are given by (see, for example, Marrelec et al. [62]):

$$\begin{array}{c} \Pi_{ij}=-\frac{C_{ij}^{-1}}{\sqrt{C_{ii}^{-1}C_{jj}^{-1}}} \end{array}$$ (20)

By inverting Eq 13 we obtain $C_{ii}^{-1}=\frac{2}{\sigma^{2}}$. When inserting this expression in Eq 20, the equivalence of partial correlation and *A* in Eq 16 is demonstrated.

#### Predicted covariance for very small values of c

When the couplings between areas are zero, the predicted covariance will be an identity matrix. Slightly increasing the value of the global coupling allows noise to diffuse from one node to its neighbors and thus the SC will show its effects. Further increases of *c* will introduce correlations among non directly connected regions.

The entries of the covariance in its diagonal basis have the form:

$$\begin{array}{c} C_{ii}^{D}\left(c\right)=-\frac{\sigma^{2}}{2}\left(\frac{1}{-1+c\lambda_{i}}\right) \end{array}$$ (21)

where *λ*_*i*_, *i* = 1, …*n* are the eigenvalues of **W**. Taylor expanding around *c*_0_ = 0:

$$\begin{array}{c} C_{ii}^{D}\left(c\right)=\frac{\sigma^{2}}{2}\left(1+\lambda_{i}c+O\left(c^{2}\right)\right) \end{array}$$ (22)

So, at the first order of approximation, a matrix proportional to the structural connectivity will be added to the identity.

#### Projectors and residuals

If {|*v*_*i*_〉} are the orthonormal eigenvectors of a symmetric matrix **A**, with relative eigenvalues *ν*_*i*_, *i* = 1, …*n*, an eigenvector’s projector will be the matrix **P**_*i*_ = |*v*_*i*_〉 〈*v*_*i*_. This projectors have the following properties:

$$\begin{array}{c} A=\sum_{i=1}^{n}\nu_{i}P_{i} \end{array}$$ (23)

$$\begin{array}{c} I=\sum_{i=1}^{n}P_{i}. \end{array}$$ (24)

If {|*u*_*i*_〉} are the orthonormal eigenvectors of the second matrix **B** and *μ*_*i*_, *i* = 1, …*n* the relative eigenvalues, we can write **B** as follows:

$$\begin{array}{ll} B & =\sum_{i=1}^{n}\mu_{i}|u_{i}\rangle \;\langle u_{i}|\\ & =\sum_{i,j,k=1}^{n}\mu_{i}|v_{j}\rangle \;\langle v_{j}|u_{i}\rangle \;\langle u_{i}|v_{k}\rangle \;\langle v_{k}|\\ & =\sum_{i,j,k=1}^{n}\mu_{i}a_{ij}a_{ik}|v_{j}\rangle \;\langle v_{k}|\\ & =\sum_{i,k=1}^{n}\mu_{i}a_{ik}^{2}P_{k}+\sum_{k\neq j;i,j,k=1}^{n}\mu_{i}a_{ij}a_{ik}|v_{j}\rangle \;\langle v_{k}| \end{array}$$ (25)

where *a*_*ij*_ = <*v*_*i*_|*u*_*i*_>. A symmetric matrix can thus be decomposed, with regards to the eigenvectors of another symmetric matrix of the same dimension, in two terms: a linear combination of the projectors of the other matrix and a residual term (obtained as a linear combination of the diadic operators, obtained from the eigenvectors, others than the projectors).

#### Projectors’ time dependent coefficients

This property can be used to decompose the sliding-windows empCov_w_ in terms of the projectors of the total empCov. A measured time-series can be written as

$$\begin{array}{c} |x\left(t\right)\rangle =\sum_{i=1}^{n}|u_{i}\rangle \;\langle u_{i}\;|\;x\left(t\right)\rangle =\sum_{i=1}^{n}b_{i}\left(t\right)\;|u_{i}\rangle . \end{array}$$ (26)

where {|*u*_*i*_〉} are now the orthonormal eigenvectors of empCov, *μ*_*i*_, *i* = 1, …*n* the relative eigenvalues and where *b*_*i*_ = <*u*_*i*_|*x*(*t*)>.

The empirical covariance of a window *w*_*k*_ of duration *w* is:

$$\begin{array}{c} C_{w_{k}}=\frac{1}{w}\int_{w_{k}}(|x\left(t\right)\rangle -|\bar{x}\rangle )\;\left(\langle x\left(t\right)|-\langle \bar{x}\left(t\right)|\right)\;dt \end{array}$$ (27)

where the bar denotes temporal averaging.

Substituting Eq (26) in Eq (27) and performing the calculation we obtain the decomposition desired:

$$\begin{array}{l} C_{w_{k}}=\\ \;=\;\sum_{i=1}^{n}\left(\frac{1}{w}\int_{w_{k}}b_{i}^{2}\left(t\right),dt-\frac{{\bar{b}}_{i}^{2}}{w^{2}}\right)|u_{i}\rangle \;\langle u_{i}|+\\ \;\sum_{i=1}\sum_{j\neq i,j=1}^{n}\left(\frac{1}{w}\int_{w_{k}}b_{i}\left(t\right)b_{j}\left(t\right),dt-\frac{{\bar{b}}_{i}{\bar{b}}_{j}}{w^{2}}\right)|u_{i}\rangle \;\langle u_{j}|. \end{array}$$ (28)

#### How variations in the structure affect the covariance

We can define *m*_*i*_ as the *n*-dimensional vector whose components are the elements of the *i*-th column of a matrix **A**, i.e.

$$\begin{array}{c} A=\left[m_{1}\;m_{2}\;...\;m_{n}\right]\;, \end{array}$$ (29)

and *v*^*i*^ as a *n*-D vector with *i*-th component equal to 1 and with all the others components null.

The matrix **A**^**′**^ derived from **A** with a small variation *ϵ* can be therefore written as

$$\begin{array}{c} A^{\prime }=\left[m_{1}\;m_{2}\;...\;\left(m_{i}+c\;\epsilon \;v^{j}\right)\;...\;\left(m_{j}+c\;\epsilon \;v^{i}\right)\;...\;m_{n}\right]\; \end{array}$$ (30)

where *c* is again the coupling parameter.

The *ij*-th component of the inverse **B**^**−1**^ of a general matrix **B** is

$$\begin{array}{c} {\left(B^{-1}\right)}_{ij}=Cof\left(B,i,j\right)\;, \end{array}$$ (31)

where *Cof*(**B**, *i*, *j*) is the determinant of the *n* − 1 × *n* − 1 sub-matrix obtained from **B** removing the *i*-th row and the *j*-th column (the minor). It follows, considering *k* ≠ *l* and (*k*, *l*) ≠ (*i*, *j*), (*j*, *i*),

$$\begin{array}{c} \begin{array}{cc} & {\left(A^{\prime -1}\right)}_{kl}=Cof\left(A^{\prime },k,l\right)\\ & =\det{\left[m_{1}\;m_{2}\;...\;\left(m_{i}+c\;\epsilon \;v^{j}\right)\;...\;\left(m_{j}+c\;\epsilon \;v^{i}\right)\;...\;m_{n}\right]}_{\backslash l}^{\backslash k}\\ & =\det{\left[m_{1}\;m_{2}\;...\;m_{i}\;...\;m_{j}\;...\;m_{n}\right]}_{\backslash l}^{\backslash k}+\\ & \;+c\;\epsilon \det{\left[m_{1}\;m_{2}\;...\;m_{i}\;...\;v^{i}\;...\;m_{n}\right]}_{\backslash l}^{\backslash k}+\\ & \;+c\;\epsilon \det{\left[m_{1}\;m_{2}\;...\;v^{j}\;...\;m_{j}\;...\;m_{n}\right]}_{\backslash l}^{\backslash k}+\\ & \;+c^{2}\;\epsilon^{2}\det{\left[m_{1}\;m_{2}\;...\;v^{j}\;...\;v^{i}\;...\;m_{n}\right]}_{\backslash l}^{\backslash k} \end{array} \end{array}$$ (32)

where ${}_{\backslash l}^{\backslash k}$ denotes that the *k*-th element of each column vectors, and the whole *l*-th vector are suppressed and where we used the multi-linearity property of the determinant. Finally, from Eq (14), we find

$$\begin{array}{c} \begin{array}{cc} -\frac{2}{\sigma^{2}}{\left(C\right)}_{kl} & ={\left(A^{-1}\right)}_{kl}\\ & =-\frac{2}{\sigma^{2}}{\left(C\right)}_{kl}+\\ & \;\;+c\;\epsilon \det{\left[m_{1}\;m_{2}\;...\;m_{i}\;...\;v^{i}\;...\;m_{n}\right]}_{\backslash l}^{\backslash k}+\\ & \;\;+c\;\epsilon \det{\left[m_{1}\;m_{2}\;...\;v^{j}\;...\;m_{j}\;...\;m_{n}\right]}_{\backslash l}^{\backslash k}+\\ & \;\;+c^{2}\;\epsilon^{2}\det{\left[m_{1}\;m_{2}\;...\;v^{j}\;...\;v^{i}\;...\;m_{n}\right]}_{\backslash l}^{\backslash k} \end{array} \end{array}$$ (33)

In the case (*k*, *l*) = (*i*, *j*) or (*k*, *l*) = (*j*, *i*) the result is trivially (**C**^**′**^)_*kl*_ = (**C**)_*kl*_.

### Analysis

Correlations used are the Pearson correlation. Due to the symmetry of the matrices analyzed we only considered the lower triangle, diagonal excluded, of each matrix when performing correlations.

P-values and confidence intervals have been evaluated through bootstrapping and, unless otherwise specified, we set alpha equal to 0.01 and performed 10000 re-samplings.

We quantified subspaces similarity through canonical correlation, i.e. the cosine of the principal angle between them. This corresponds to the scalar product for 1 dimensional subspaces and, in general, ranges from 1 for high similarity to 0 for low resemblance.

### Non-linear modeling

The non-linear model chosen to compare the resulting FC with our analytical one, is the reduced version of the Wong-Wang mean field model presented in [14, 16].

In this model neurons, described by the classical Integrate-and-Fire model, are aggregated to form excitatory or inhibitory populations, depending on their synaptic receptor type (AMPA and NMDA for the former and GABA-A for the latter). Each cortical area is then modeled as a fully connected recurrent network of one excitatory and one inhibitory population. Mean field approximations techniques are applied to each cortical area which allow to describe its mean neural activity *x*_*i*_ through a single one dimensional equation. The dynamics of the full brain network can be described by the following set of coupled nonlinear stochastic differential equations

$$\begin{array}{c} \frac{dS_{i}\left(t\right)}{dt}=-\frac{S_{i}}{\tau_{S}}+\left(1-S_{i}\right)\gamma H\left(x_{i}\right)+\sigma \nu_{i}\left(t\right) \end{array}$$ (34)

$$\begin{array}{c} H\left(x_{i}\right)=\frac{ax_{i}-b}{1-exp(-d\left(ax_{i}-b\right))} \end{array}$$ (35)

$$\begin{array}{c} x_{i}=wJ_{N}S_{i}+cJ_{N}\sum_{j}W_{ij}S_{j}+I_{0} \end{array}$$ (36)

Where *W*_*ij*_ is the SC, *c* the global coupling parameter, *H*(*x*_*i*_) and *S*_*i*_ represent the population firing rate and the average synaptic gating variable. We set all the parameters value as in [14] except for the noise amplitude: *w* = 0.9 for the local excitatory recurrence; *a* = 270(*n*/*C*), *b* = 108(*Hz*), *d* = 0.154(*s*) for the input-output function; *γ* = 0.641/1000, *τ*_*S*_ = 100*ms* for the kinetic parameters; synaptic coupling was set at *J*_*N*_ = 0.2609(*nA*) and the overall effective external input at *I*_0_ = 0.3(*nA*). The noise was set to *σ* = 0.00005 unless otherwise specified.

We generated the simulated time-series through stochastic Euler integration using an integration step of 0.1 ms.

## Supporting Information

S1 Fig Effect of the diagonal in the parameter exploration. FCs are represented for four values of the parameter: when *c* is close to zero the off-diagonal entries of FC reflect SC. When increasing the global coupling, correlations emerge that do not merely reflect structure. We represent the same four matrices with (top row) and without (third row) the main diagonal in order to show how maintaining the diagonal during the parameter exploration helps to find the right scaling between variances and covariances. Below each FC matrix we show a plot with the entries of the empFC on the x axis and the corresponding value in aFC on the y axis. For each plot we also report the global correlation between aFC and empFC. It is possible to note that the peak in correlation for the ‘with diagonal’ condition is different than that for the ‘without diagonal’ one.
(TIF)

S2 Fig Analytic versus empirical SC. The main difference between aSC and empSC is in the inter-hemispherical connections. To better appreciate the predictive power of the model for the two cases, we can divide the figures of aSC and empSC in their intra-hemispherical and inter-hemispherical parts. We can see that the presence of a strong second diagonal in aSC introduces the different scaling of the the empirical and analytical connections (as observed comparing Fig 2 bottom left and bottom center).
(TIF)

S3 Fig Sliding-windows analysis of SC. We performed a sliding-windows analysis of the time-series and computed each window’s empCov_w_. We then applied the analytical operators to obtain SC. The left panel of the figure shows the aSC for the whole time-series (average across subjects), the right panel, instead, displays the STD across window’s aSC (averaged across subjects). Low standard deviation results for connections having a stable contribution to function, a more strongly fluctuating contribution instead gives high standard deviation.
(TIF)

S1 Folder Data and scripts. The folder contains the data used in this work together with the scripts necessary to perform the analysis.
(ZIP)
