# Supplementary figures and images for: Analytical Operations Relate Structural and Functional Connectivity in the Brain
Source: PLoS One. 2016 Aug 18;11(8):e0157292. doi: 10.1371/journal.pone.0157292 (PMC4990451; doi:10.1371/journal.pone.0157292)

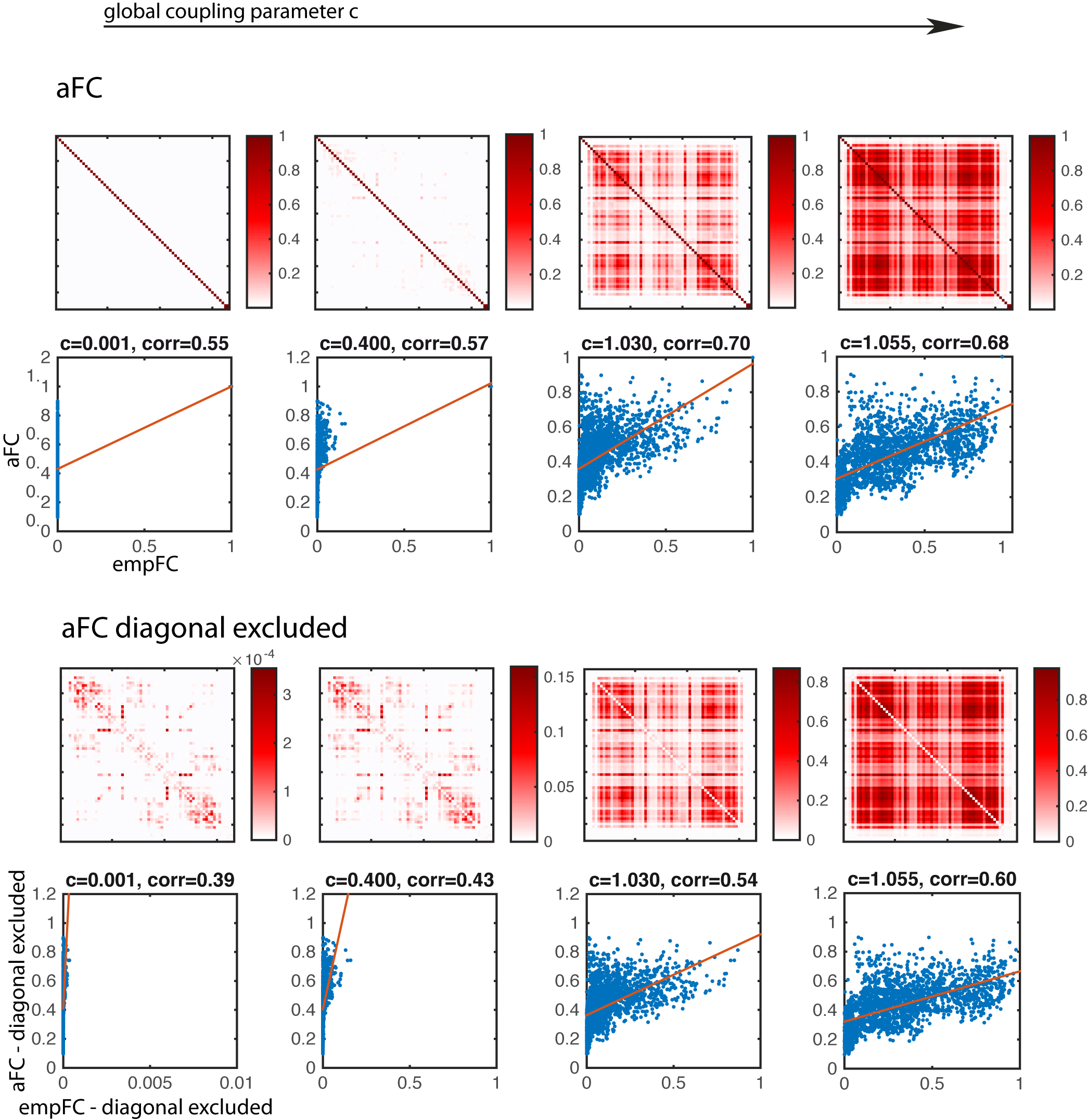

Supplement: S1 Fig — FCs are represented for four values of the parameter: when c is close to zero the off-diagonal entries of FC reflect SC. When increasing the global coupling, correlations emerge that do not merely reflect structure. We represent the same four matrices with (top row) and without (third row) the main diagonal in order to show how maintaining the diagonal during the parameter exploration helps to find the right scaling between variances and covariances. Below each FC matrix we show a plot with the entries of the empFC on the x axis and the corresponding value in aFC on the y axis. For each plot we also report the global correlation between aFC and empFC. It is possible to note that the peak in correlation for the ‘with diagonal’ condition is different than that for the ‘without diagonal’ one. (TIF) [file pone.0157292.s001.tif]

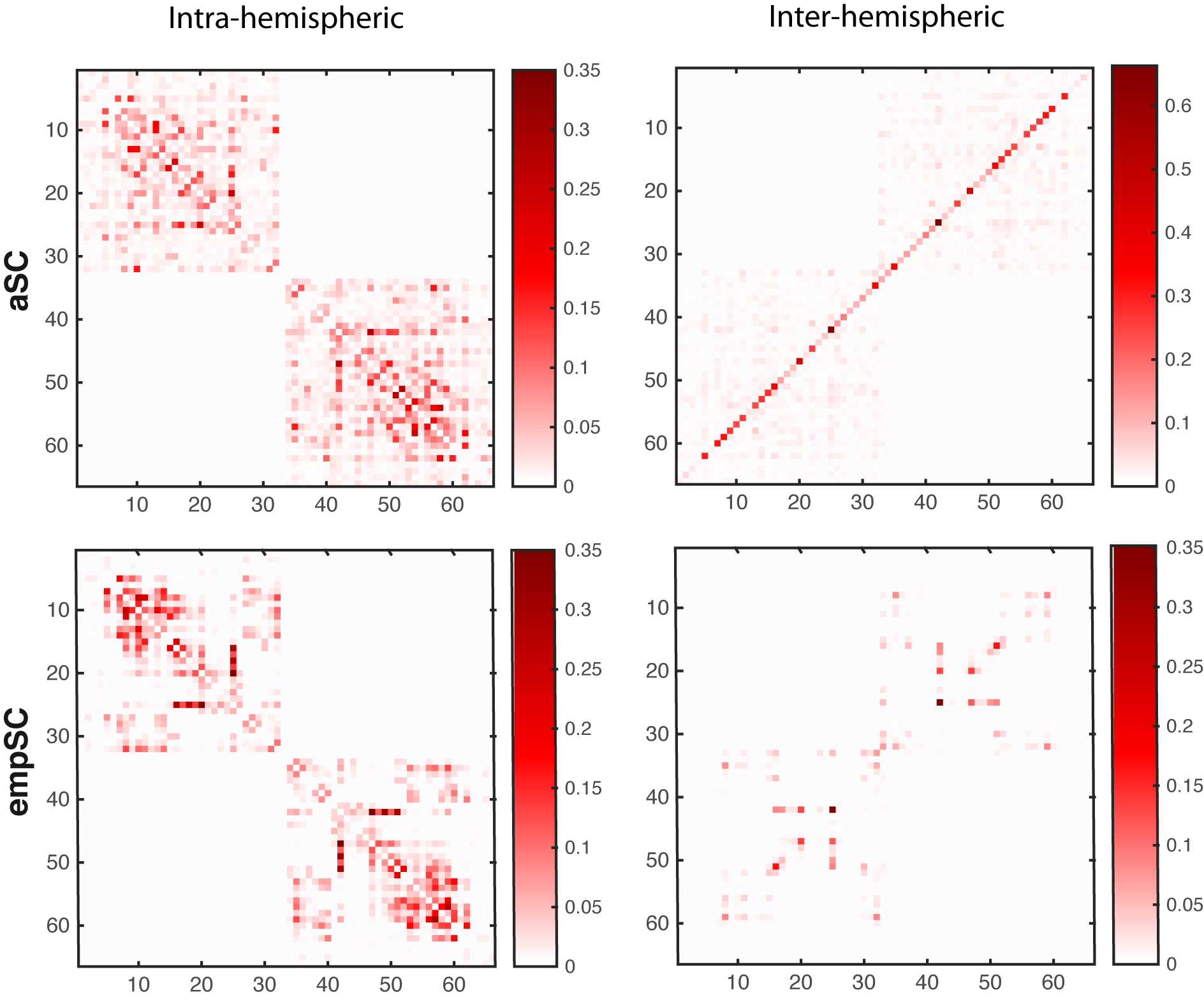

Supplement: S2 Fig — The main difference between aSC and empSC is in the inter-hemispherical connections. To better appreciate the predictive power of the model for the two cases, we can divide the figures of aSC and empSC in their intra-hemispherical and inter-hemispherical parts. We can see that the presence of a strong second diagonal in aSC introduces the different scaling of the the empirical and analytical connections (as observed comparing Fig 2 bottom left and bottom center). (TIF) [file pone.0157292.s002.tif]

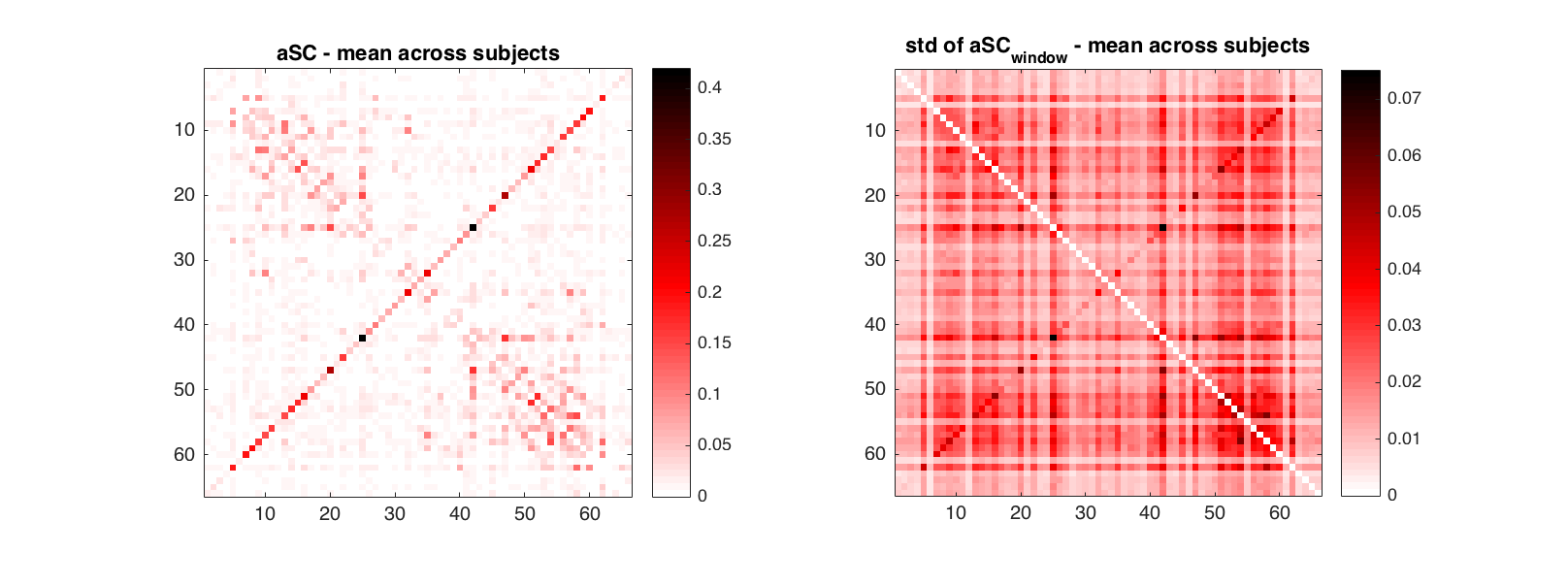

Supplement: S3 Fig — We performed a sliding-windows analysis of the time-series and computed each window’s empCovw. We then applied the analytical operators to obtain SC. The left panel of the figure shows the aSC for the whole time-series (average across subjects), the right panel, instead, displays the STD across window’s aSC (averaged across subjects). Low standard deviation results for connections having a stable contribution to function, a more strongly fluctuating contribution instead gives high standard deviation. (TIF) [file pone.0157292.s003.tif]
